# Distribution and phylogenetic diversity of *Anopheles* species in malaria endemic areas of Honduras in an elimination setting

**DOI:** 10.1186/s13071-020-04203-1

**Published:** 2020-07-01

**Authors:** Denis Escobar, Krisnaya Ascencio, Andrés Ortiz, Adalid Palma, Gustavo Fontecha

**Affiliations:** Microbiology Research Institute, Universidad Nacional Autónoma de Honduras, Tegucigalpa, Honduras

**Keywords:** *Anopheles* spp., Phylogeny, *cox*1, ITS2, Honduras

## Abstract

**Background:**

*Anopheles* mosquitoes are the vectors of malaria, one of the most important infectious diseases in the tropics. More than 500 *Anopheles* species have been described worldwide, and more than 30 are considered a public health problem. In Honduras, information on the distribution of *Anopheles* spp. and its genetic diversity is scarce. This study aimed to describe the distribution and genetic diversity of *Anopheles* mosquitoes in Honduras.

**Methods:**

Mosquitoes were captured in 8 locations in 5 malaria endemic departments during 2019. Two collection methods were used. Adult anophelines were captured outdoors using CDC light traps and by aspiration of mosquitoes at rest. Morphological identification was performed using taxonomic keys. Genetic analyses included the sequencing of a partial region of the cytochrome *c* oxidase 1 gene (*cox*1) and the ribosomal internal transcribed spacer 2 (ITS2).

**Results:**

A total of 1320 anophelines were collected and identified through morphological keys. Seven *Anopheles* species were identified. *Anopheles albimanus* was the most widespread and abundant species (74.02%). To confirm the morphological identification of the specimens, 175 and 122 sequences were obtained for *cox*1 and ITS2, respectively. Both markers confirmed the morphological identification. *cox*1 showed a greater nucleotide diversity than ITS2 in all species. High genetic diversity was observed within the populations of *An. albimanus* while *An. darlingi* proved to be a highly homogeneous population. Phylogenetic analyses revealed clustering patterns in *An. darlingi* and *An. neivai* in relation to specimens from South America. New sequences for *An. crucians*, *An. vestitipennis* and *An. neivai* are reported in this study.

**Conclusions:**

Here we report the distribution and genetic diversity of *Anopheles* species in endemic areas of malaria transmission in Honduras. According to our results, both taxonomic and molecular approaches are useful tools in the identification of anopheline mosquitoes. However, both molecular markers differ in their ability to detect intraspecific genetic diversity. These results provide supporting data for a better understanding of the distribution of malaria vectors in Honduras.
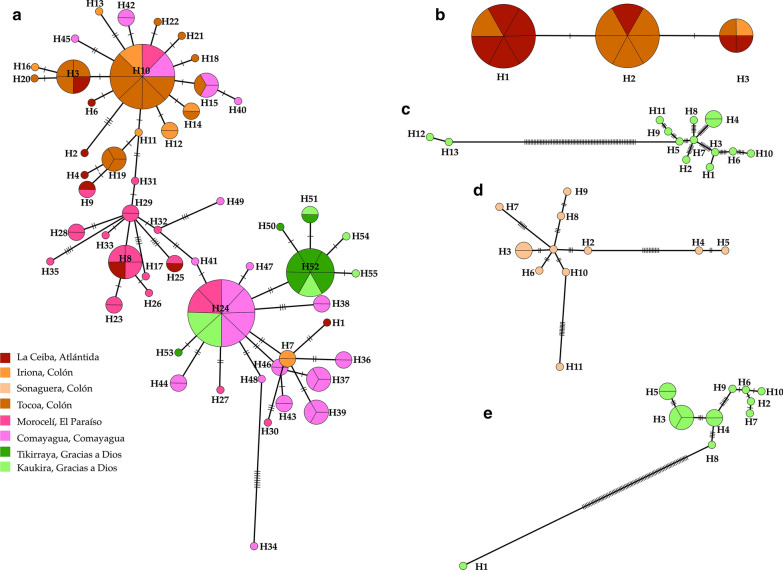

## Background

According to the World Health Organization (WHO), more than 228 million cases of malaria occurred worldwide in 2018. The WHO Region of the Americas accounted for less than 0.5% of all malaria cases. A decrease in the number of malaria cases has been recorded in many endemic countries of the continent, except mainly in Venezuela, Brazil and Colombia [1]. Nine countries in Central America and Hispaniola are taking part in a sub-regional initiative to eliminate malaria over the next years [2]. As a signatory to this agreement, Honduras has managed to reduce vectorial transmission by more than 96% since 2004, reporting only 651 cases in 2018 [1]. This reduction can be attributed in part to the integrated control of *Anopheles* species.

The genus *Anopheles* includes more than 500 formally recognized species and several unclassified members (*incertae sedis*), some of them grouped into species complexes [3]. Based on molecular markers such as ITS2, both dominant vector species (DVS) and secondary vectors of malaria in the Americas are grouped into three sub-genera: *Anopheles* (*Anopheles*); *An*. (*Nyssorhynchus*); and *An*. (*Kerteszia*) [4, 5]. Approximately 70 species of these three sub-genera are capable of transmitting malaria parasites [6], and of those, 30 to 40 have sufficient vector capacity to be considered as public health problems [7, 8]. There are discrepancies in the literature with regard to the number of dominant *Anopheles* species in Mesoamerica. According to a global map of dominant malaria vectors published in 2012, there are at least seven species reported on the isthmus [9]. *Anopheles pseudopunctipennis* and *An. albimanus* are the most prevalent species, whereas *An. darlingi* shows more focalized distribution patterns. *Anopheles aquasalis* is predominant in the coastal areas of southern Central America and with lower vector capacity [10]. Other authors point out that the most relevant species of malaria vectors recognized in Mesoamerica are *An. albimanus*, *An*. *pseudopunctipennis*, *An*. *darlingi*, *An*. *vestitipennis* and *An*. *punctimacula* [2].

Scientific information regarding malaria vector species in Honduras is scarce. The first partial record of anophelines in the country dates from 1930, when Dr Antonio Vidal described seven *Anopheles* species from four ecological regions [11]. Vidal’s report was followed by a brief description in 1998 of the local species on the island of Utila (Bay Islands) [12]. Additionally, some specimens of anophelines collected in Honduras and other countries have been used in order to determine their genetic diversity [13]. Other authors have described extensively the composition of *Anopheles* species in the Neotropics [14], or have made notable efforts to predict the distribution of the DVS of malaria in the Americas through intensive literature searches and an evidence-based approach [9, 10]. Despite these efforts, there are still important information gaps about *Anopheles* species in Honduras, and the only verifiable data on their distribution in the country are internal reports by the Ministry of Health, which publishes them as part of routine entomological surveillance since 2013. According to those reports, 12 species of anophelines have been identified through morphometric keys: *Anopheles albimanus*; *An. albitarsis*; *An. apimacula*; *An. argyritarsis*; *An. crucians*; *An. darlingi*; *An. gabaldoni*; *An. grabhami*; *An. neomaculipalpus*; *An. pseudopunctipennis*; and *An. punctimacula*. Another information gap in Honduras is the lack of molecular data that support the classification of mosquitoes based on morphometric keys. Molecular markers are critical to distinguish between evolutionarily close or cryptic species, even using immature specimens [15, 16].

To optimize the limited resources available for vector control strategies in Honduras, it is necessary to know in depth the distribution of anophelines considered vectors of malaria. This study aims to provide an update on the diversity of the *Anopheles* mosquitoes in Honduras, supporting its distribution in morphological data, as well as in two molecular markers.

## Methods

### Study sites

Entomological captures were carried out in 10 sites located in 8 municipalities in 5 departments of the country (Atlántida, Colón, Comayagua, El Paraíso and Gracias a Dios) from February to October 2019 (Table 1). All the capture sites were located near small rural villages in which agricultural and fishing activities take place. Active foci of malaria were reported during 2018 at each site [1]. The departments of Atlántida, Colón and Gracias a Dios are classified as very humid tropical and coastal ecosystems less than 550 m above sea level (masl), while Comayagua and El Paraíso are considered as subtropical with heights above 550 masl and drier ecosystems. The average temperature varies between 25 °C and 33 °C, and the relative humidity ranges from 40% to 91% in all sites depending on the season of the year. The population’s livelihood in the selected areas is mainly based on agricultural and livestock activities. The study sites are those monitored by the Ministry of Health of Honduras to undertake routine entomological surveillance as they remain endemic to malaria by *Plasmodium vivax*. Malaria due to *P. falciparum* malaria is reported almost exclusively in Gracias a Dios. Geographical coordinates and altitude of the collection sites are shown in Table 1.

**Table 1:** *Anopheles* specimen collection sites

Department	Municipality	Coordinates	Altitude (masl)	Month of collection
Atlántida	La Ceiba	15.748587, − 86.900546	7	February
Atlántida	La Ceiba	15.758790, − 86.867092	7	February
Colón	Iriona	15.938416, − 85.058888	4	March
Colón	Iriona	15.773889, − 85.134556	27	March
Colón	Sonaguera	15.629846, − 86.287587	82	April
Colón	Tocoa	15.655448, − 86.04725	38	April
El Paraíso	Morocelí	14.103168, − 86.917882	600	August
Comayagua	Comayagua	14.439279, − 87.689953	588	August
Gracias a Dios	Tikirraya	15.018379, − 83.641264	13	October
Gracias a Dios	Kaukira	15.309131, − 83.565868	8	October

### Mosquito collection

Mosquito catches were performed for one night at each collection site. Atlántida and Colón were visited during the dry season of the year (February to April), and El Paraíso, Comayagua and Gracias a Dios were visited in the rainy season (August to October) (Table 1). Two collection methods were used at each site to capture the greatest amount and diversity of *Anopheles* species. The first method used outdoor CDC traps only fitted with light as an attractant, with 3 to 5 traps per site in a period from 18:00 to 6:00 h. Traps were separated a minimum of 50 meters from each other. Traps were placed in the outdoor structures of the houses where humans reside and also in structures where domestic animals rest. The second method was by aspiration of mosquitoes resting outdoors, during the period from 18:00 to 21:00 h [17]. After collection, mosquitoes identified as anopheline were placed on a Petri dish with silica gel and transported at room temperature to the laboratory in Tegucigalpa where they were stored at − 20 °C until later morphological identification [18].

### Morphological identification

After collection, all specimens were classified by sex and identified morphologically under a stereoscope using keys for anophelines of Central America and Mexico [19]. After morphological identification, wing and leg vouchers of each specimen were preserved as a reference in the Center for Genetic Research of the National Autonomous University of Honduras. Each mosquito was then stored individually at − 20 °C for subsequent molecular tests.

### *cox*1 gene

A subset of morphologically identified specimens were chosen randomly for molecular analysis. For the most common *Anopheles* species, between 8% and 17% of the specimens were selected to proportionally represent all the capture sites. For less common species, at least 50% of the specimens were selected for sequencing.

DNA was extracted from each specimen according to the protocol provided by the AxyPrep MAG Tissue-Blood gDNA Kit, Axygen® (Corning Incorporated, Life Sciences, Tewksbury, MA, USA). Preliminarily, the mosquitoes were macerated with a pestle in a 1.5 ml conical tube together with 50 µl of lysis solution provided by the kit. DNA was stored at − 20 °C until further use. Molecular analyses were performed on *Anopheles* mosquitoes to confirm species and calculate genetic variation within species. Two molecular markers were used: cytochrome *c* oxidase 1 gene (*cox*1), and the internal transcribed spacer 2 (ITS2). The following primers were used to amplify a fragment of *cox*1: LCO1490 (5′-GGT CAA CAA ATC ATA AAG ATA TTG G-3′) and HCO2198 (5′-TAA ACT TCA GGG TGA CCA AAA ATC A-3′) [20]. Reactions were carried out in a volume of 50 µl, with 25 µl of Taq Master Mix 2× (Promega, Madison, Wisconsin, USA), 2.0 µl of each primer (10 µM), 2 µl of acetylated bovine albumin (BSA) (10 mg/ml), 4 µl of DNA, and nuclease-free water. The PCR program was as follows: 1 cycle at 95 °C for 10 min, 37 cycles at 94 °C for 1 min, 48 °C for 1 min, 72 °C for 1 min, and 1 cycle at 72 °C for 7 min.

**Fig. 1:**
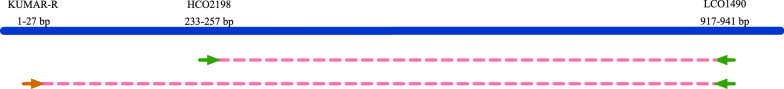
Scheme of the region of the *cox*1 gene amplified. Target sites of the primers used in the PCR are indicated by arrows

Some mosquito specimens that could not be amplified with the pair of primers described above were amplified using LCO1490 and a reverse primer described by Kumar et al. [21] (5′-AAA AAT TTT AAT TCC AGT TGG AAC AGC-3′; Fig. 1), with the following reagents and concentrations: 25 µl of Taq Master Mix 2× (Promega); 1 µl of each primer (10 µM); 2 µl of DNA; and 21 µl of nuclease-free water. The cycling conditions were: 1 cycle at 95 °C for 5 min, 5 cycles at 94 °C for 40 s, 45 °C for 1 min, and 1 cycle at 72 °C for 1 min, 37 cycles at 94 °C for 1 min, 54 °C for 1 min, 72 °C for 90 s and a final extension step at 72 °C for 10 min. The PCR products were separated by electrophoresis in 1% agarose gels with ethidium bromide.

### ITS2 ribosomal region

For ITS2 amplification, PCR reactions were performed using the universal primers [22]: 5.8S (5′-ATC ACT CGG CTC GTG GAT CG-3′) and 28S (5′-ATG CTT AAA TTT AGG GGG TAG TC-3′). Reagent concentrations were as follows: 25 µl of Taq Master Mix 2× (Promega), 2 µl of each primer 10 µM, 2 µl of DNA, and water for a total reaction volume of 50 µl. PCR amplifications were performed with the following conditions: 94 °C for 2 min, 34 cycles of 94 °C for 30 s, 57 °C for 30 s, 72 °C for 30 s, and a final extension step at 72 °C for 10 min.

### Sequence analysis

The amplification products of both *cox*1 and ITS2 markers were sequenced on both strands using the same primers used for the PCR. A representative subset of mosquitoes of all species and all collection sites was selected for sequencing. Purification and sequencing services were provided by Psomagen (https://www.macrogenusa.com). The sequences were edited with the Geneious® 9.1.7 software (Biomatters Ltd., Auckland, New Zealand) and were deposited in two databases: Barcode of Life Data System (BOLDSYSTEMS; http://www.boldsystems.org), and in the National Center for Biotechnology Information (NCBI; https://www.ncbi.nlm.nih.gov). Barcode index numbers (BINs) and accession numbers were obtained for each sequence. All sequences were submitted as queries to NCBI through the BLAST tool [23] under default parameters to identify the most similar sequences in the GenBank nucleotide collection.

### Nucleotide diversity (π) and number of haplotypes

In order to calculate the nucleotide diversity (π), the sequences of both molecular markers were analysed separately and by species. The sequences were aligned using the MUSCLE algorithm. MEGA v10.0 software [24] with 1000 bootstrap replicates was used to calculate the pairwise distance using the Maximum Composite Likelihood substitution method, and 95% as the site coverage cut-off. The percentage of identical bases for each species and between species was calculated in order to demonstrate the reported “barcoding gap”, which is the difference between inter- and intraspecific genetic distances within a group of organisms.

The haplotype diversity was calculated with *R* through the function *hap.div* of *pegas* (v0.12 package) and using the Nei and Tajima’s method [25]. Haplotype frequencies were calculated using the *Haplotype* function with default parameters, and the haplotype network was computed with the *haploNet* function using an infinite site model, pairwise deletion missing data, and probability of parsimonious link [26].

### Phylogenetic analysis

Nucleotide sequences were trimmed and manually corrected using the Geneious® 9.1.2 software (https://www.geneious.com). The ClustalW tool was used to align sequences. Phylogenetic trees were constructed using the Tamura-Nei distance model, the Neighbor-Joining method and a bootstrap of 1000 replicates with no outgroup. Length, identical sites and pairwise % identity were calculated for each molecular marker and each species.

To calculate the phylogenetic relationships between specimens collected in Honduras with those collected in other countries of the Americas, analogous *cox*1 and ITS2 sequences for all available *Anopheles* species were downloaded from the GenBank database. Sequences were aligned and phylogenetic trees constructed under the same parameters described above.

## Results

### Distribution of *Anopheles* species

Eight municipalities (10 collection sites) were visited to collect anopheline mosquitoes. A total of 1320 adult individuals of 7 *Anopheles* species were collected and identified using a taxonomic key: *Anopheles* (*Nyssorhynchus*) *albimanus* Wiedemann; *An.* (*Nys.*) *darlingi* Root; *An.* (*Anopheles*) *vestitipennis* Dyar & Knab; *An.* (*An*.) *crucians* Wiedemann; *An.* (*An.*) *pseudopunctipennis* Theobald; *An.* (*An.*) *punctimacula* (*s.l*.) Dyar & Knab; and *An.* (*Kerteszia*) *neivai* Howard, Dyar & Knab (Table 2). More morphological details of the vouchers can be observed in the project “CIGAN Bionomy of *Anopheles* spp. in Honduras” of the BOLD database.

**Table 2:** Distribution of *Anopheles* species identified using taxonomic keys according to capture site and geographical region

Department	Location	*An. albimanus*	*An. darlingi*	*An. vestitipennis*	*An. crucians*	*An. pseudopunctipennis*	*An. punctimacula*	*An. neivai*	Total (%)
Atlántida	La Ceiba 1	307	61	1	–	–	1	8	378 (28.64)
Atlántida	La Ceiba 2	21	17	2	–	–	–	–	40 (3.03)
Colón	Iriona 1	7	–	–	–	2	–	–	9 (0.68)
Colón	Iriona 2	8	–	–	–	–	–	–	8 (0.60)
Colón	Sonaguera	–	–	–	–	10	–	–	10 (0.76)
Colón	Tocoa	96	14	–	–	–	1	–	111 (8.41)
El Paraíso	Morocelí	23	–	–	–	–	–	–	23 (1.7)
Comayagua	Comayagua	294	–	–	–	1	–	–	295 (22.34)
Gracias a Dios	Tikirraya	44	–	–	–	–	–	–	44 (3.30)
Gracias a Dios	Kaukira	177	–	92	132	–	–	1	403 (30.50)
Total		977 (74.02%)	92 (6.97%)	95 (7.20%)	132 (10.0%)	13 (0.98%)	2 (0.015%)	9 (0.07%)	1320 (100)

**Fig. 2:**
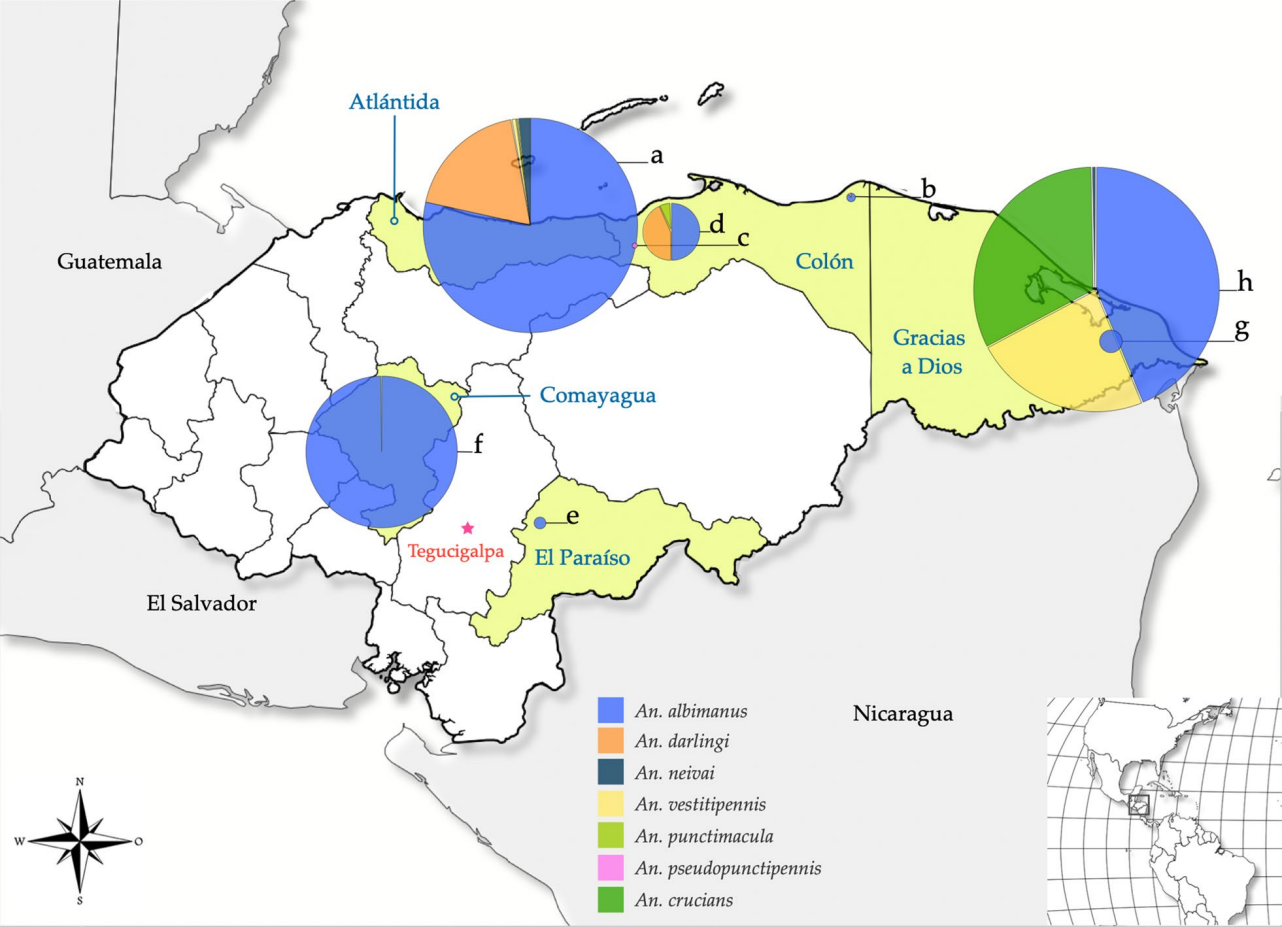
Map of Honduras showing eight collection sites. The pie charts show the proportion of *Anopheles* species collected at each site. The size of the charts is proportional to the number of specimens collected. **a** La Ceiba (Atlántida). **b** Iriona (Colón). **c** Sonaguera (Colón). **d** Tocoa (Colón). **e** Morocelí (El Paraíso). **f** Comayagua (Comayagua). **g** Tikirraya (Gracias a Dios). **h** Kaukira (Gracias a Dios)

Most specimens were identified as *Anopheles albimanus* (74.02%), *An. crucians* (10%), *An. vestitipennis* (7.2%), and *An. darlingi* (6.97%). The remaining 3 species accounted for less than 1% of the total. *Anopheles albimanus* was found in all locations except Sonaguera (Colón). The highest species richness (*n* = 5) was found in La Ceiba (Atlántida) followed by Kaukira (Gracias a Dios) (*n* = 4). Moreover, in 5 other localities only one to 3 species were recorded. *Anopheles crucians* was only found in Gracias a Dios. The greatest mosquito abundance was obtained in Gracias a Dios (33.8%), Atlántida (31.67%), and Comayagua (22.34%) (Fig. 2). *An. darlingi* was only present in Atlántida and Colón.

### Nucleotide sequences

A total of 160 *cox*1 sequences and 122 ITS2 sequences were obtained for 6 out of 7 *Anopheles* species. These sequences proportionally represent the geographical origin of each mosquito species. No sequences of *An. neivai* were obtained for either of the two markers. A second set of primers for *cox*1 (Fig. 1) was enabled generation of 5 sequences for *An. neivai* and 10 sequences for 4 other species: *An. albimanus*; *An. darlingi*; *An. punctimacula*; and *An. vestitipennis*.

All *cox*1 and ITS2 sequences were deposited in the BOLD system database and the following BINs were assigned: CIGAN001-19 to CIGAN067-19, CIGAN068-20 to CIGAN178-20. These sequences were also deposited in GenBank under the following accession numbers: MT033921-MT034050; MT040803-MT040831; MT048394-MT048399; MT049952-MT049958; MT053086; MT062520; MT066404; MN998028-MN998149.

The *cox*1 intra- and interspecific percentage of identity for the 6 species were non-overlapping, averaging 99.04% (98.35–100%) and 88.52% (86.51–91.60%), respectively. Inter-specific pairwise genetic distances greater than 3% support the “barcoding gap” between the *Anopheles* species reported in this study.

*Cox*1 sequences were analysed with the NCBI BLAST tool in order to confirm the morphological identification of the species. *Anopheles albimanus*, *An. darlingi*, *An. pseudopunctipennis* and *An. punctimacula* were correctly identified by BLAST with identity percentages of 95.6–99.7%. Sequences of *An. crucians*, *An. vestitipennis* and *An. neivai* could not be identified by BLAST due to the lack of sequences for these species in the databases, making them the first *cox*1 sequences reported for the three species on GenBank. All species were correctly identified by ITS2 with identity percentages of 99.63–100% except for *An. vestitipennis*, due to lack of sequences for this species in the databases. This is also the first report of ITS2 sequences for *An. vestitipennis*. In summary, the morphological identification coincided with the molecular identification based on both markers for the species with sequences available in the databases.

### Nucleotide diversity and haplotypes

**Fig. 3:**
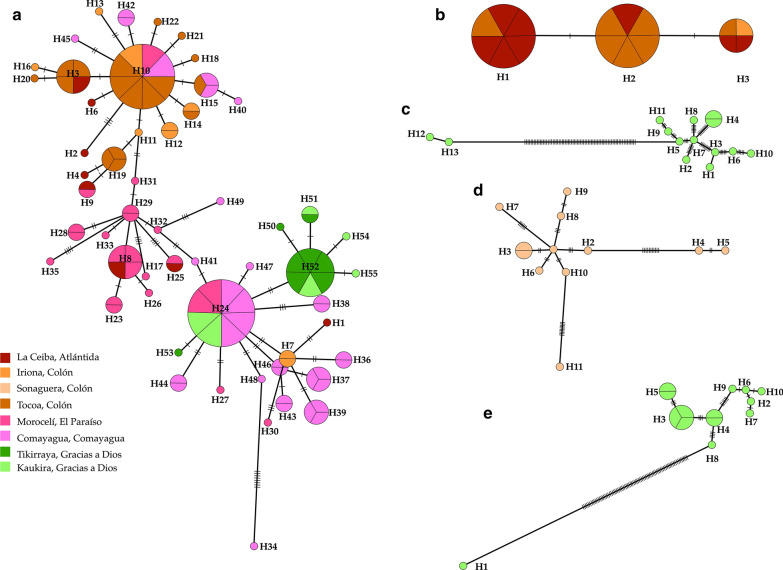
*Cox*1 haplotype networks for *Anopheles* spp. collected in eight locations of Honduras. **a***An. albimanus*. **b***An. darlingi*. **c***An. crucians*. **d***An. pseudopunctipennis*. **e***An. vestitipennis*

Intraspecific variation was calculated for both markers. *Cox*1 showed a higher level of polymorphism than ITS2. According to *cox*1, the species with the highest nucleotide diversity was *Anopheles crucians* (π = 0.05), followed by *An. vestitipennis* (π = 0.03) (Table 3). The species with the lowest diversity was *Anopheles darlingi. Anopheles albimanus* revealed a high number of haplotypes (*n* = 55). *Anopheles pseudopunctipennis* showed the highest proportion of *cox*1 haplotypes with respect to the number of sequences analysed (11/11) and *An. darlingi* revealed the lowest haplotype index (3/16). ITS2 showed a low number of haplotypes (1–4) in all species (Table 3) (Fig. 3).

**Table 3:** Intraspecific comparison of nucleotide sequences and number of haplotypes for *cox*1 and ITS2 in 5 species of *Anopheles* of Honduras

Marker	*An. albimanus*	*An. crucians*	*An. darlingi*	*An. pseudopunctipennis*	*An. vestitipennis*
*cox*1					
Length	712	711	684	684	681
*n*	103	14	16	11	14
Identical sites	659	600	682	654	596
Identical sites (%)	92.6	85.3	99.7	95.6	87.5
Pairwise % identity	99.1	95.8	99.9	98.9	97.7
π	0.01	0.05	0.00	0.01	0.03
No. of haplotypes	55	13	3	11	10
Haplotypes/N	0.53	0.93	0.19	1.0	0.71
ITS2					
Length	566	380	596	567	576
*n*	76	13	10	7	14
Identical sites	552	367	593	549	567
Identical sites (%)	97.7	99.5	99.5	97.0	98.6
Pairwise % identity	99.9	99.9	99.9	99.1	99.7
π	0.0	0.0	0.0	0.0	0.0
No. of haplotypes	3	1	1	3	4
Haplotypes/N	0.04	0.08	0.1	0.43	0.29

### Phylogenetic analysis

**Fig. 4:**
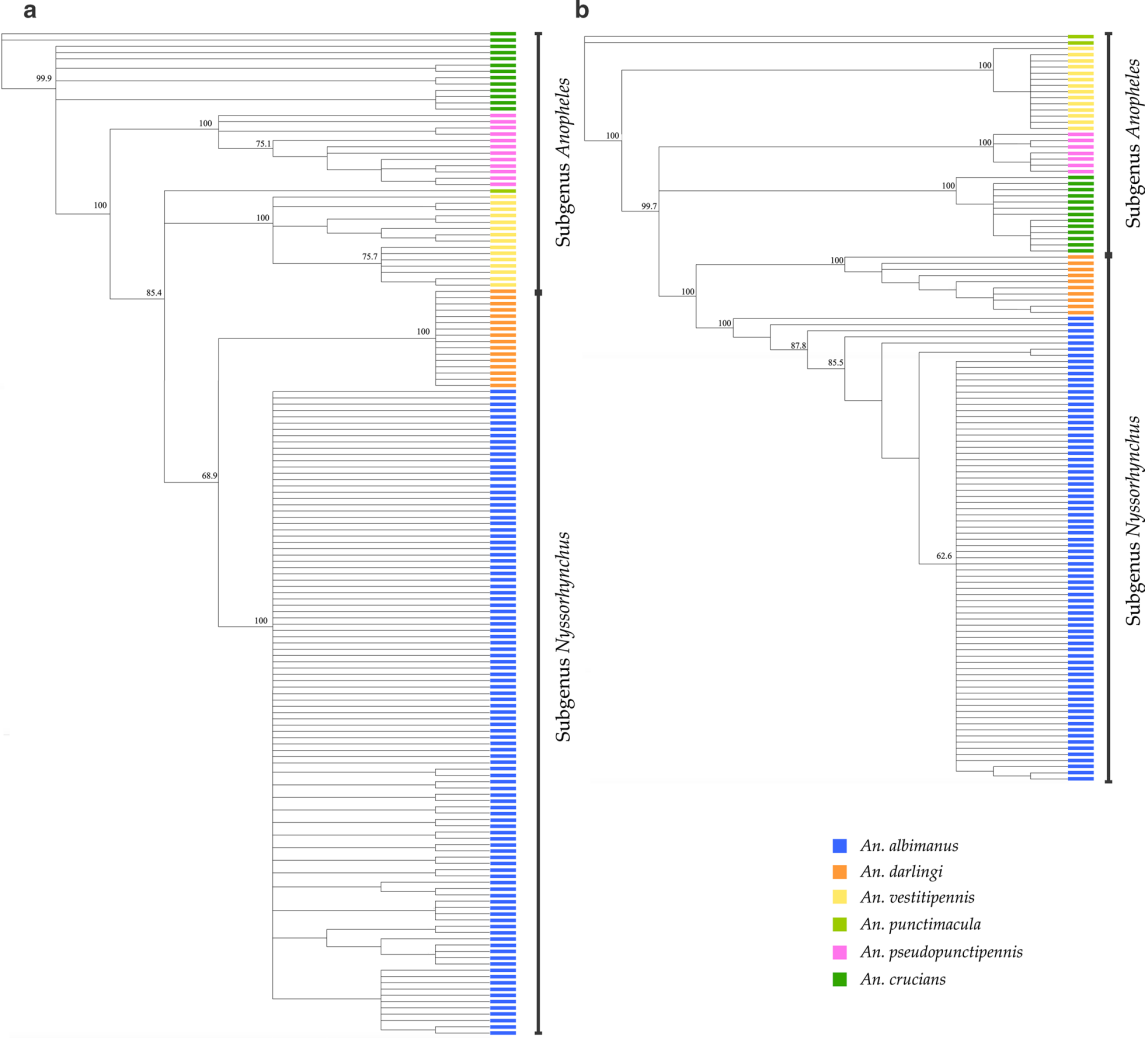
Phylogenetic analysis of *cox*1 (**a**) and ITS (**b**) sequences of six *Anopheles* species. Dendrograms were constructed using the Neighbor-Joining method and the Geneious 9.1.7 software with a bootstrap of 1000 replicates

Three analyses were performed to infer phylogenetic relationships between sequences. The first analysis included all the sequences of each marker for six *Anopheles* species. Both dendrograms (*cox*1 and ITS2) showed that the species clearly separated into clades (Fig. 4).

The second analysis included sequences of *An. albimanus* classified according to geographical region. Phylogenetic relationships based on *cox*1 sequences showed only one separate cluster that included 11 out of 14 sequences of mosquitoes collected in Gracias a Dios. The other sequences were not clustered (Additional file 1: Figure S1). ITS2 sequences did not reveal any clustering according to geographical origin. This analysis was not performed for other *Anopheles* species due to the low intraspecific variation.

The third phylogenetic analysis included the *cox*1 sequences of five species obtained in this study (*An. albimanus*, *An. darlingi*, *An. pseudopunctipennis*, *An. punctimacula* and *An. neivai*) together with analogous sequences available in GenBank in order to understand the relationships between individuals from Honduras and mosquitoes from other countries in the Neotropical region. The same analysis was performed separately with the ITS2 sequences of five species from Honduras (*An. albimanus*, *An. darlingi*, *An. pseudopunctipennis*, *An. punctimacula* and *An. neivai*) and sequences from specimens from other countries.

**Fig. 5:**
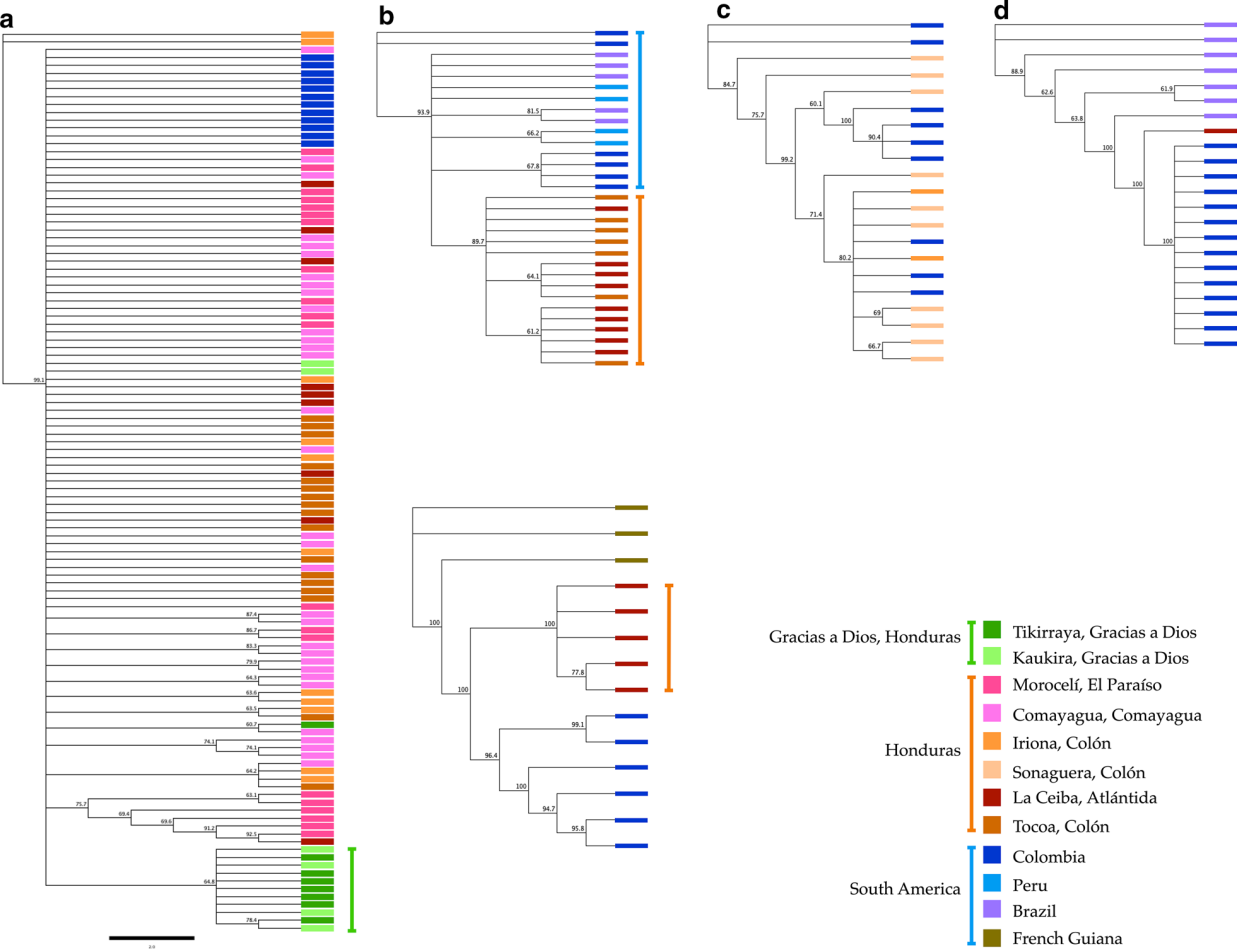
Phylogenetic analysis of the *cox*1 gene of *Anopheles* spp. from Honduras and four South American countries. Coloured boxes indicate the geographical region where the insects were captured. **a***Anopheles albimanus.***b***An. darlingi.***c***An. pseudopunctipennis.***d***An. punctimacula.***e***An. neivai*. Trees were constructed using the Neighbor-Joining method and the Geneious 9.1.7 software with a bootstrap of 1000 replicates

The phylogenetic tree of *An. albimanus* included 12 *cox*1 sequences of mosquitoes from Colombia and 103 sequences of mosquitoes from Honduras; however, the sequences from Colombia clustered together with the majority of sequences from Honduras. Eleven sequences of mosquitoes captured in Gracias a Dios formed a well-supported clade (Fig. 5a). For *An. darlingi* 16 sequences from Honduras, 6 sequences from Colombia, 5 sequences from Brazil, and 4 sequences from Peru were analysed. According to this analysis the population was divided into two clusters, one including all the sequences of Honduras, and another with the sequences from South America (Fig. 5b).

In addition, 12 sequences of *An. pseudopunctipennis* from Honduras and 9 sequences from Colombia were analysed. For the analysis of *An. punctimacula*, 7 sequences from Brazil, 14 sequences from Colombia and 1 sequence from Honduras were included. No clusters were detected for both species (Fig. 5c, d). The analysis for *An. neivai* included 3 sequences from French Guiana, 6 sequences from Colombia, and 5 sequences from Honduras. The specimens of the three countries showed a defined separation according to geographical origin (Fig. 5e).

The phylogenetic analysis of the ITS2 sequences included data for a total of 8 countries of the Americas, including Honduras, Colombia, Brazil, French Guiana, Panama, Nicaragua, Ecuador and Belize. None of the trees could demonstrate separation of populations based on geographical origin (Additional file 2: Figure S2).

## Discussion

This study provides updated information on the distribution and genetic diversity of *Anopheles* species in endemic malaria regions of Honduras. Seven *Anopheles* species were found. *Anopheles albimanus* was the most common species and the most widely distributed. This is consistent with the existing literature. *Anopheles albimanus* has been described as the dominant species in Central America, the Caribbean and some coastal regions of northern South America [9, 13, 14]. This has been demonstrated through studies conducted in Colombia [27], Panama [28], Belize [29] and Guatemala [30]. The predominance of this species, considered as a generalist species, can be attributed to the wide range of habitats, feeding preferences, and heights mosquitoes can tolerate [31]. In this study, mosquitoes were collected at eight municipalities. In seven sites, *An. albimanus* was the most frequently captured species despite the ecological differences between all locations. La Ceiba (Atlántida) and Kaukira (Gracias a Dios) yielded greater species richness (*n* = 4–5), similar to reports from Cordoba, in the coastal region of the Colombian Caribbean [27]. This high richness could be influenced by the high temperatures of the coastal regions, high relative humidity and the presence of permanent larval habitats.

The second most abundant species collected in Kaukira was *Anopheles crucians*. This finding is remarkable since this species was not registered anywhere else in this study. *Anopheles crucians* has been recognized as one of the five most important malaria vectors in the Honduras [32], and has been reported as one of the most frequent species in Belize, Guatemala, Honduras and Nicaragua [29, 33]. Since La Mosquitia is the main region with permanent transmission and the highest number of malaria cases in the country throughout the year, it would be interesting to further explore the importance of this species in the malaria transmission. On the other hand, *An. darlingi* was collected only in two coastal departments (Atlántida and Colón), consistent with previous reports [34]. This species is known for its preference to inhabit areas of high rainfall and where the tropical forest is close to the ocean [14, 35].

Sequences of the *cox*1 gene and the ITS2 ribosomal region were obtained for the seven *Anopheles* species that were identified morphologically. Four and six species of anophelines were identified by BLAST of the *cox*1 and ITS2 sequences, respectively. Up to the moment of the analysis, no analogous sequences of *cox*1 for *Anopheles crucians*, *An. vestitipennis* and *An. neivai* were available in the databases. There were no sequences of ITS2 for *An. vestitipennis* in the GenBank database either. Consequently, these would be the first sequences reported.

The barcoding approach compares an individual sequence with a reference library to uniquely identify an organism to a species. Thus, our findings support the barcoding method as a useful tool to confirm the correct assignment of misidentified or unidentified *Anopheles* species using morphology [27, 36–38]. When comparing the individual ability of both markers to identify or confirm *Anopheles* species in Honduras, it seems that both are informative enough and fulfil their purpose [38, 39]. Some authors report problems to solve and identify species when these markers are used individually [40] and suggest that a multilocus approach might have a greater power of discrimination [41, 42]. However, our study shows that both molecular markers are useful separately and are a good complement to the identification of *Anopheles* based on taxonomic keys [43].

Intraspecific variation was calculated for five *Anopheles* species. A greater nucleotide diversity (π) and number of haplotypes with *cox*1 than with ITS2 were observed. According to this result, *cox*1 would be more informative to decipher the intraspecific phylogenetic relationships. Some authors reported different findings when analysing the phylogeny of the *Anopheles* Hyrcanus Group using ITS2 sequences downloaded from GenBank. They concluded that ITS2 would be more reliable than *cox*1 as a phylogenetic marker among very close taxa. This discrepancy may be attributed to the fact that the Hyrcanus Group includes at least 25 species widely distributed in a large geographical area [15, 16]. Discrepancies between markers are expected since there are different evolutionary processes that act differently on mitochondrial and nuclear genes [44]. Nevertheless, *cox*1 can be considered a more useful marker for evidencing intraspecific genetic diversity between *Anopheles* spp. in Honduras.

The species with the lowest genetic diversity was *An. darlingi* based on 16 *cox*1 sequences analysed. Although the number of sequences studied is low, it is possible to say that the population is relatively homogeneous. High homogeneity within the population could be attributed to the fact that the geographical area in which the mosquitoes were collected was small or to the capture of siblings. Similar results were reported in a study conducted in Darien, at the border between Panama and Colombia, with 40 individuals who showed low nucleotide diversity (π = 0.0006) [45].

On the other hand, when the phylogenetic relationships of *Anopheles darlingi* specimens collected in the Caribbean of Honduras was analysed together with 15 sequences obtained from mosquitoes from Colombia, Peru and Brazil, the resulting Neighbor-Joining tree showed two well-differentiated clades between the populations of South America and the population of Honduras. This could support the theory of geographical and reproductive isolation between the populations of northern Central America and South America. There are several studies that analyze the population continuity of *An. darlingi* throughout Central and South America. Several researchers report that *An. darlingi* populations in Central and South America reveal significant differences through the use of morphological and behavioural markers [46], RAPDs [47], *cox*1 [48], and microsatellite loci [49]. It has been hypothesized that this geographical isolation could be attributed to the absence or low population densities of *An. darlingi* in Nicaragua and Costa Rica [10, 45]. However, more information is needed in this regard to generate a robust hypothesis of reproductive isolation for *An. darlingi*.

*Anopheles neivai* was the second species that showed well-separated clades within the dendrogram. One clade included five sequences from Honduras, a second clade included three sequences from French Guiana, and a third clade consisted of six sequences from Colombia. A recent study analysed four mitochondrial and ribosomal sequences of 35 specimens from Guatemala, Panama, and the southern Pacific coast of Colombia. Phylogenetic networks showed two clusters well differentiated by geography [50]. Although the authors concluded that their results support the existence of a single taxonomic entity, sequences from Guatemala clearly separated from those of the rest of Panama and Colombia. This result is consistent with the findings of our study and supports the hypothesis of the existence of two possible entities: *An. neivai* (*sensu stricto*) in South America, and *An. neivai* “A” in Central America [50].

Phylogenetic analyses and haplotype networks for *An. albimanus* detected 55 haplotypes without any clustering pattern based on geographical origin. This suggests high genetic diversity and the existence of gene flow between populations. This finding suggests that there is no evidence of isolation that could lead to the generation of divergent lineages in *An. albimanus*. The only lineage that showed a low to moderate bootstrap support (64.8) was composed of 11 sequences of mosquitoes collected in La Mosquitia. This result is interesting given that this region is socially isolated from the rest of the country by the Río Plátano biosphere reserve. However, this hypothetical isolation should be confirmed in the future by more robust and informative molecular markers such as microsatellite loci [51]. Future sampling should also include specimens from other geographical regions, particularly from the Honduras islands in the Caribbean.

## Conclusions

In this study, the distribution and genetic diversity of some *Anopheles* species in malaria endemic areas of Honduras have been described through a morphological approach and two molecular markers. Conventional taxonomy and *cox*1 and ITS2 sequencing proved to be useful tools for the correct identification of anopheline species. However, both molecular markers differ in their ability to detect intraspecific genetic diversity. According to phylogenetic analyses, the only two species that seem to show some level of structuring with respect to South American lineages are *An. darlingi* and *An. neivai*. *Anopheles albimanus* was the most abundant and widely distributed species and there is no evidence of disruption in gene flow between populations of different geographical areas. In summary, our results contribute to the development of a sequence-based confirmation tool for anopheline identification in Honduras, which is an important step for the monitoring and integrated control of malaria vectors. Future work should be aimed at a wider sampling of other geographical regions and in the use of microsatellite markers to assess the population structure of these anopheline species.

## Supplementary information

**Additional file 1: Figure S1.** Phylogenetic analysis based on *cox*1 (**a**) and ITS sequences (**b**) of *Anopheles albimanus*. Coloured boxes indicate the geographical region where the insects were captured. Dendrograms were constructed using the Neighbor-Joining method and the Geneious 9.1.7 software with a bootstrap of 1000 replicates.

**Additional file 2: Figure S2.** Phylogenetic analysis based on the ITS2 region of rDNA for *Anopheles* spp. from Honduras and other seven countries. Coloured boxes indicate the geographical region where the insects were captured. **a***Anopheles albimanus*. **b***An. darlingi*. **c***An. pseudopunctipennis*. **d***An. punctimacula*. **e***An. crucians*. Trees were constructed using the Neighbor-Joining method and the Geneious 9.1.7 software with a bootstrap of 1000 replicates.

## Data Availability

Data supporting the conclusions of this article are included within the article and its additional files. The newly generated sequences were submitted to the GenBank database under the accession numbers MT033921-MT034050; MT040803-MT040831; MT048394-MT048399; MT049952-MT049958; MT053086; MT062520; MT066404; MN998028-MN998149. The raw data used and/or analysed during the present study are available from the corresponding author on reasonable request.

## Abbreviations

WHO: World Health Organization
*cox*1: Cytochrome *c* oxidase subunit 1
ITS: Ribosomal internal transcribed spacer
CDC: Centers for Disease Control and Prevention, USA
PCR: Polymerase chain reaction
BINs: Barcode index numbers
BOLD: Barcode of Life Data System
NCBI: National Center for Biotechnology Information
BLAST: Basic Local Alignment Search Tool
Masl: Meters above sea level
RAPDs: Random amplified polymorphic DNA
